# A Giant Atherosclerotic Ascending Aortic Aneurysm (13.5 cm) With Arch Involvement Successfully Managed by Valve-Sparing Total Arch Replacement: A Case Report

**DOI:** 10.7759/cureus.117433

**Published:** 2026-10-04

**Authors:** Randa N Tabbah, Jacques Irani, Sam Barbar, Maroun Khattar, Karam Karam

**Affiliations:** 1 Cardiology, Centre Hospitalier Universitaire Notre Dame des Secours, Byblos, LBN; 2 Cardiology, Lebanese American University, Beirut, LBN; 3 Cardiology, University of Balamand, Beirut, LBN; 4 Cardiothoracic Surgery, Centre Hospitalier Universitaire Notre Dame des Secours, Byblos, LBN

**Keywords:** aortic surgery, ascending aortic aneurysm, atherosclerosis, case report, giant thoracic aneurysm, total arch replacement

## Abstract

Giant ascending aortic aneurysms (AscAAs), conventionally defined as aneurysms exceeding 10 cm in diameter, are exceedingly rare in contemporary practice due to earlier diagnosis and prophylactic surgical intervention. AscAAs of atherosclerotic origin are particularly uncommon because proximal thoracic aortic aneurysms are typically associated with medial degeneration, connective tissue disorders, or bicuspid aortic valve disease rather than advanced atherosclerosis. Reports of aneurysms exceeding 13 cm remain exceptional in the literature.

We report the case of a 70-year-old woman with multiple cardiovascular risk factors, including heavy tobacco exposure and type 2 diabetes mellitus, who presented with progressive dyspnea, constitutional symptoms, and hemodynamic compromise. Computed tomography angiography demonstrated a giant AscAA measuring 13.5 cm with extension into the aortic arch (8.7 cm), associated with periaortic fluid and imaging features suggestive of impending rupture. The aortic root and descending thoracic aorta were spared. Emergency open surgical repair was successfully performed using supracoronary ascending aortic replacement, total arch replacement under deep hypothermic circulatory arrest, and separate supra-aortic branch reconstruction with a branched Dacron graft. Histopathology confirmed an atheromatous aneurysm with thrombotic degeneration, while microbiological studies excluded infection.

This case highlights the extreme rarity of massive atherosclerotic AscAAs and demonstrates that successful treatment is achievable through advanced, anatomy-driven open surgical reconstruction. Complete valve-sparing total arch replacement with separate branch vessel reconstruction provided durable exclusion of extensive disease while preserving native root structures. This report adds to the limited literature on extreme-diameter ascending aneurysms and underscores the importance of prompt diagnosis and individualized operative planning.

## Introduction

Thoracic aortic aneurysm is defined as a permanent localized dilation of the aorta exceeding 50% of its normal diameter. In women, the normal ascending aortic diameter is approximately 2.8-3.0 cm, making the present case’s 13.5-cm aneurysm nearly five times the normal size [1,2]. Current international guidelines [3] recommend surgical intervention for ascending aortic aneurysms (AscAAs) at diameters ≥5.5 cm in most patients because of sharply increasing risks of rupture, dissection, and death.

Giant AscAAs >10 cm are extraordinarily uncommon. Most contemporary cases are identified before reaching this threshold because of improved imaging surveillance and earlier operative referral. Furthermore, while atherosclerosis is a predominant cause of aneurysmal disease in the descending thoracic and abdominal aorta, the ascending aorta is relatively resistant to isolated atherosclerotic degeneration due to distinct embryological and structural properties. Consequently, giant atherosclerotic AscAAs are exceptionally rare [4,5].

In this report, we present a case of one of the largest reported atherosclerotic AscAAs, successfully treated with valve-sparing total arch replacement with separate branch reconstruction.

## Case presentation

A 70-year-old woman presented to the Emergency Department with a one-week history of progressive severe dyspnea (New York Heart Association Class IV), generalized fatigue, anorexia, weight loss, and nocturnal diaphoresis. Her past medical history included type 2 diabetes mellitus, a heavy smoking history (>50 pack-years), hypothyroidism following thyroidectomy, and anxiety with a depressive disorder.

On presentation, the patient was hemodynamically fragile, with hypotension and significant respiratory distress. Cardiovascular examination demonstrated regular heart sounds with a small aortic murmur. Initial laboratory testing revealed elevated inflammatory markers and mild anemia (Table 1).

**Table 1:** Laboratory findings Reference range: WBC 4.5-10.5, Neutrophil 35-65, Lymphocytes 23-53, Hemoglobin 13-17, Hematocrit 40-54, Platelet count 140-400, CRP 0-5, Na 136-145, K 3.5-5.1, Ca 8.8-10.2, Mg 1.4-2.7, Cl 98-107, Urea 17-46, Creatinine 0.8-1.2.

Complete blood count		Reference range
WBC	8,120 cells/µL	4.5-10.5
Neutrophils	80.40%	35-65
Lymphocytes	16.1%	23-53
Hemoglobin	10.5 g/dL	13-17
Hematocrit	32.9%	40-54
Platelet Count	391,000 /µL	140-400
Biochemistry		
C-Reactive Protein (CRP)	231.3 mg/L	0-5
Sodium (Na)	142 mEq/L	136-145
Potassium (K)	3.87 mEq/L	3.5-5.1
Calcium (Ca)	8.70 mg/dL	8.8-10.2
Magnesium (Mg)	1.78 mg/dL	1.4-2.7
Chloride (Cl)	110 mEq/L	98-107
Urea (BUN)	85 mg/dL	17-46
Creatinine	1.43 mg/dL	0.8-1.2
Liver Function Tests	Within normal limits	-

A contrast-enhanced chest computed tomography (CT) revealed an AscAA of 13.5 cm maximal diameter and an aortic arch aneurysm of 8.7 cm. Moreover, a periaortic fluid collection was noticed with an intra-aneurysmal air bubble, a radiological concern for fissuration or contained rupture. In addition, a normal aortic root diameter of 24 mm was seen with a preserved descending thoracic aorta (Figure 1).

**Figure 1:**
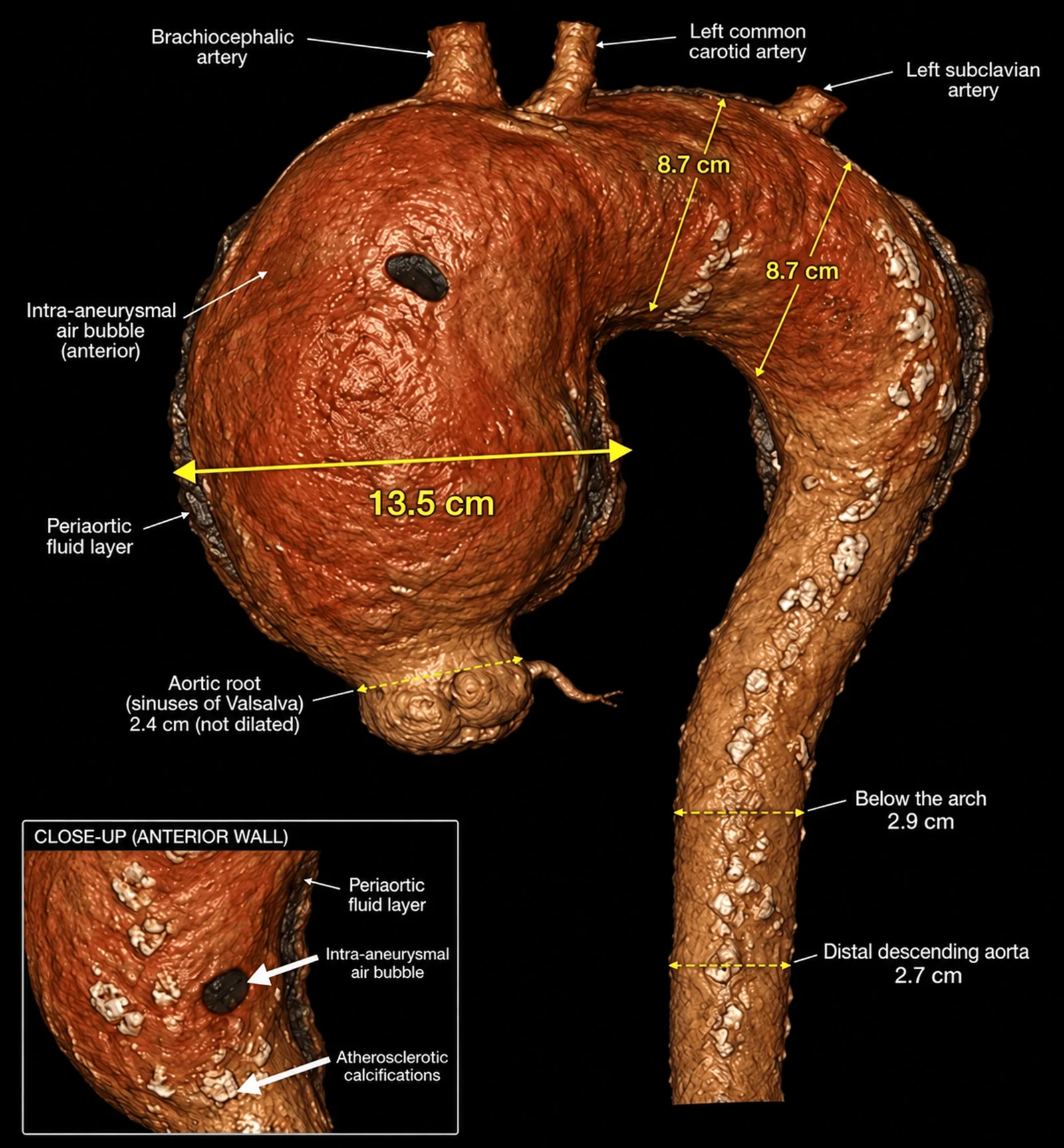
A 3D reconstruction of the proximal thoracic aortic aneurysm

Transthoracic echocardiography demonstrated a preserved left ventricular ejection fraction, mild aortic regurgitation with a tricuspid aortic valve, and severe ascending aortic dilation (13 cm). Given the aneurysm’s extreme size, symptomatic presentation, and impending rupture risk, urgent surgery was undertaken.

Following systemic heparinization and confirmation of an adequate activated clotting time, cardiopulmonary bypass was established through left femoral arterial and venous cannulation using 17-Fr and 24-Fr cannulas, respectively. A median sternotomy was performed, and the AscAA was dissected while the patient was cooled to 18°C. The aneurysmal ascending aorta and arch were resected, exposing the sinotubular junction proximally, the descending thoracic aorta distal to the left subclavian artery distally, and the three supra-aortic vessel stumps. The native aortic valve and coronary ostia were preserved. Cardioplegia was administered directly through the coronary ostia at 15-minute intervals.

Supracoronary ascending aortic and total arch replacement were performed using a 32-mm woven polyester graft (InterGard) to which three separate 8-mm branches had been anastomosed before implantation. Reconstruction began with the distal graft-to-descending-aorta anastomosis under deep hypothermic circulatory arrest, using 3-0 polypropylene sutures reinforced with Teflon felt. After completion of the distal anastomosis, systemic perfusion was resumed and rewarming initiated, with the proximal graft and its branches clamped. The proximal graft anastomosis was then completed at the sinotubular junction using 3-0 polypropylene sutures with external Teflon felt reinforcement. The clamp was subsequently removed to restore myocardial perfusion.

During supra-aortic vessel reconstruction, selective antegrade cerebral perfusion was delivered directly through cannulas placed in the brachiocephalic and left common carotid arteries and secured with umbilical tape snares. The supra-aortic vessels were individually anastomosed to the graft branches using 5-0 polypropylene sutures in the following order: left subclavian artery, left common carotid artery, and brachiocephalic artery. Following de-airing, completion of rewarming to 37°C, and confirmation of hemostasis, the patient was weaned from cardiopulmonary bypass without complications. The native descending aorta was retained beyond the distal anastomosis (Figure 2).

**Figure 2:**
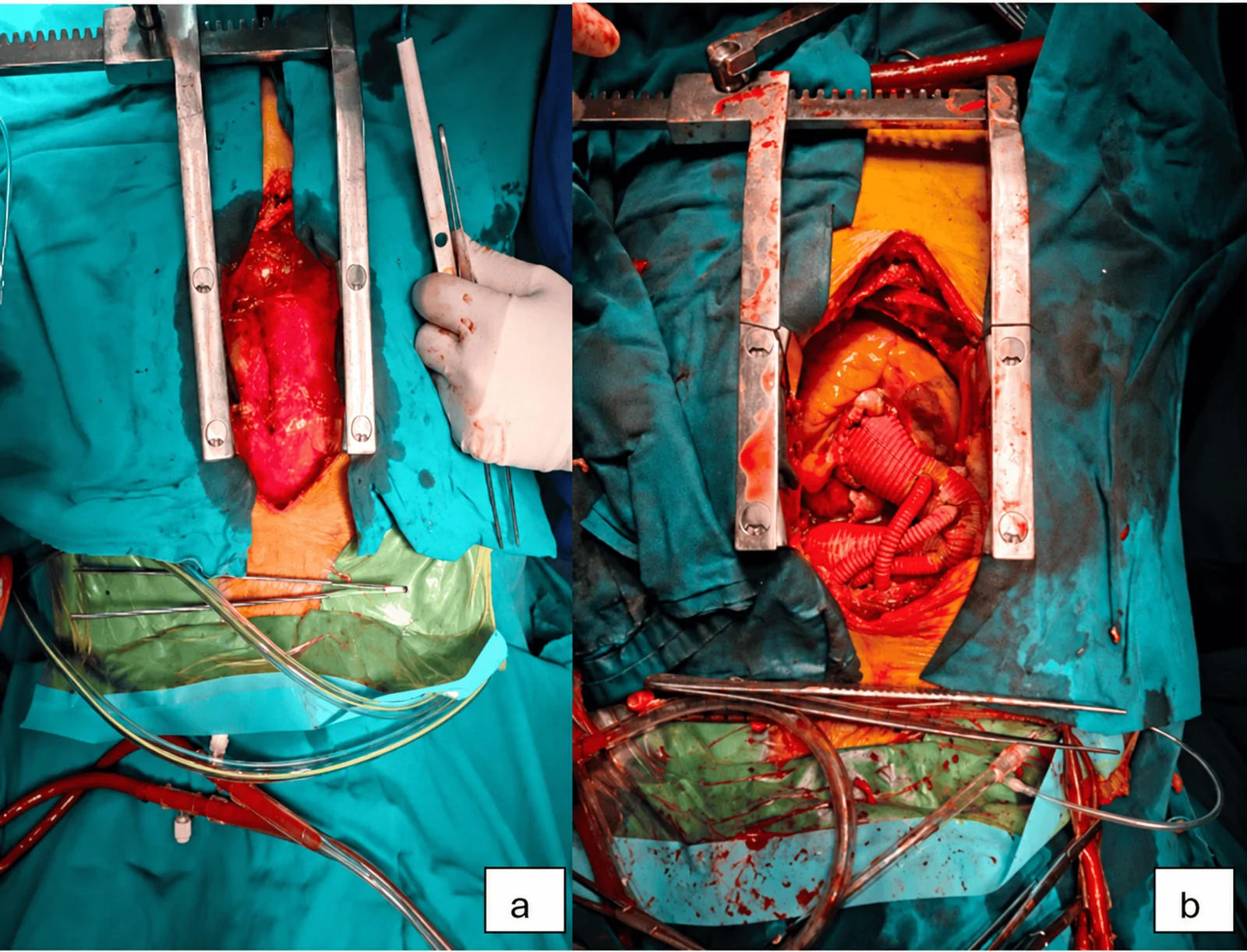
Intraoperative images a) Ascending aortic aneurysm seen through the sternotomy incision; b) Aortic graft replacement surgery with reconstruction of the brachiocephalic artery, left common carotid artery, and left subclavian artery using a branched graft technique.

The operative times were as follows: a cross-clamp time of 35 minutes, a total circulatory arrest time of 49 minutes, and a cardiopulmonary bypass time of 109 minutes. The carotid perfusion time was approximately two hours. Histopathological findings demonstrated atheromatous aneurysmal degeneration with semi-recent fibrin-clot thrombosis, with no evidence of infection or malignancy. The postoperative course was favorable. The patient recovered without major complications or neurological deficits. She was discharged in stable condition six days postoperatively.

## Discussion

This case is notable for two principal reasons: the extraordinary aneurysm dimension and the complexity of the surgical management.

Regarding the extreme rarity of the aneurysm size, AscAAs larger than 10 cm are exceptionally uncommon, and cases exceeding 13 cm are among the rarest described in the published literature [4]. Davies et al. demonstrated that aneurysms exceeding 6 cm carry a markedly elevated annual risk of rupture or dissection, making survival to 13.5 cm highly unusual [6].

Several features distinguish this case; the most interesting is that the diameter is nearly fivefold greater than normal female ascending aortic dimensions. Moreover, histological evaluation confirmed an atherosclerotic etiology despite the proximal location. The extensive arch involvement, with preserved root and descending thoracic aorta, is also a specific finding in this case. Furthermore, the symptomatic but non-catastrophic initial presentation despite advanced pathology is also surprising. The ascending aorta’s relative resistance to atherosclerotic aneurysm formation makes this pathology particularly unusual. While age and conventional vascular risk factors may contribute, the case remains an extraordinary outlier compared with typical patterns of proximal thoracic aneurysm [1].

Because the aneurysm involved both the ascending aorta and transverse arch, isolated ascending replacement would have been inadequate. Complete resection of diseased tissue was necessary to minimize future rupture, recurrent aneurysm formation, and residual pathology.

The advantages of separate branch reconstruction, compared with en bloc island reimplantation, may include more complete exclusion of diseased arch tissue, reduced retained native aortic wall, greater long-term durability, and improved anatomical customisation in distorted giant aneurysm anatomy. This approach is particularly advantageous in patients with a massive arch aneurysm burden, severe atherosclerotic degeneration, friable tissue, and the need for maximal pathological tissue excision [7,8]. Published series by Kazui, Spielvogel et al., and LeMaire et al. support branched graft approaches in complex arch pathology, particularly when long-term durability supersedes modest increases in operative complexity [9-11].

Rationale against frozen elephant trunk: The frozen elephant trunk or classic elephant trunk procedures are primarily indicated when distal arch or descending thoracic pathology is present. In this patient, the descending thoracic aorta was normal; therefore, additional distal intervention would have introduced unnecessary procedural complexity without clear anatomical benefit [12].

This case demonstrates that even extreme ascending aneurysms with arch extension can be successfully managed through individualized, aggressive open surgical repair when performed with careful anatomical planning. Preservation of the native valve and root while replacing all diseased ascending and arch tissue represented an optimal balance between radical pathology exclusion and physiological preservation.

## Conclusions

This case represents an exceptionally rare giant atherosclerotic AscAA measuring 13.5 cm, among the most extreme proximal thoracic aneurysms reported in modern literature. Its significance lies in the extraordinary anatomical magnitude, highlighting the rare natural history required for survival to such dimensions. The advanced surgical management demonstrates that valve-sparing total arch replacement with separate supra-aortic branch reconstruction can provide effective and durable treatment for highly complex proximal aortic disease. Continued reporting of such rare cases is important to expand the evidence base regarding optimal operative strategies for extreme AscAAs.
